# Explainable Deep Learning Model for Predicting Overall Survival in Patients Receiving Palliative Radiotherapy for Bone Metastases

**DOI:** 10.3390/curroncol33090528

**Published:** 2026-09-02

**Authors:** Yui Watanabe, Takuya Tomoda, Akiko Iwata, Hirokazu Matsuno, Hiroto Hayakawa, Takeshi Nagata

**Affiliations:** Department of Radiology, Daiyukai General Hospital, Ichinomiya 491-8551, Japan

**Keywords:** deep learning, prognosis, radiotherapy, bone neoplasms, radiation dosage

## Abstract

**Purpose:** Although machine learning-based prediction of overall survival (OS) in palliative radiotherapy for bone metastases has been investigated, explainable deep learning (DL) models remain underexplored. This study aimed to develop and validate an explainable DL model to predict OS in this setting, and to examine whether this flexible model provides predictive value beyond a standard Cox model based on routinely collected baseline variables. **Methods and Materials:** We analyzed all 472 eligible patients who received palliative radiotherapy for bone metastases between January 2013 and August 2024; patients alive with less than one year of follow-up were retained as right-censored observations. The primary endpoint was OS over a fixed 1-year horizon. A DeepSurv model using 14 baseline predictors, including the planned prescribed dose (biologically effective dose, BED10), was developed with repeated 5-fold cross-validation (K = 5, R = 10) and compared with standard and ridge-penalized Cox models fitted on identical splits. Performance was assessed by the time-dependent concordance index (C-index), integrated Brier score (IBS), time-dependent area under the curve (AUC) at 90, 180, and 365 days, and a calibration analysis at one year; 95% confidence intervals (CI) were obtained by patient-level bootstrapping of the pooled out-of-fold predictions. Shapley Additive Explanations (SHAP) and SurvLIME were computed on the held-out test sets. **Results:** Within one year, 242 patients (51.3%) died; median OS was 225 days (95% CI: 189–287). The DeepSurv model achieved a pooled time-dependent C-index of 0.779 (95% CI: 0.751–0.807), an IBS of 0.135 (95% CI: 0.122–0.149), and AUCs of 0.892 (0.857–0.925), 0.862 (0.822–0.895), and 0.856 (0.814–0.895) at 90, 180, and 365 days, with an observed/expected ratio of 0.94 and a calibration slope of 1.02; discrimination was comparable to the Cox model (C-index 0.763, 95% CI: 0.737–0.789). SHAP identified poor performance status as the dominant predictor (mean |SHAP| 0.178), followed by male sex (0.067), high-risk primary tumor type (0.063), multiple bone metastases (0.047), and planned dose (0.033), the latter being the only leading feature associated with lower predicted mortality; SurvLIME gave consistent results. In multivariable Cox analysis, performance status (hazard ratio [HR] 2.21 per standard deviation [SD], *p* < 0.001) and planned dose (HR 0.71 per SD, *p* < 0.001) were independently associated with OS. **Conclusions:** The explainable DL model predicted OS after palliative radiotherapy for bone metastases with discrimination and calibration comparable to those of a well-specified Cox model, and its feature attributions agreed with the Cox coefficients, suggesting that the prognostic information in these baseline variables is essentially additive and can therefore be delivered at the bedside as a simple score, without dedicated AI infrastructure and without loss of predictive performance. The combined use of SHAP and SurvLIME verified that the model relies on established clinical factors, most prominently performance status, and provides patient-level explanations. Pending external validation, such prediction may support individualized decisions on treatment goals and radiation schedules.

## Simple Summary

Predicting overall survival after palliative radiotherapy for bone metastases plays a critical role in determining appropriate treatment strategies, including adjusting treatment intensity according to individual patient prognosis. Although machine learning-based survival prediction has been investigated in this setting, explainable deep learning models remain underexplored. Prognostic scores used in this setting add up risk points for individual factors, but whether this simple additive form misses prognostic information had not been tested. This study developed and evaluated an explainable deep learning model, which can capture more complex patterns, using data from 472 patients. The model predicted survival during the first year after radiotherapy with discrimination and calibration comparable to those of a Cox proportional hazards model, indicating that this prognostic information can be provided as a simple bedside score rather than requiring artificial intelligence software. The explainability analyses identified poor performance status as by far the strongest predictor, followed by male sex, high-risk primary tumor type, the burden of metastatic disease, and the planned radiation dose; a higher planned dose was the only leading factor associated with lower predicted mortality. These findings support explainable, individualized survival estimation as an aid to decisions on treatment goals and radiation schedules.

## 1. Introduction

The skeleton is a common site of distant cancer metastases, ranking third in frequency after the lungs and liver [1,2,3]. Metastatic lesions most often involve the vertebrae, pelvis, ribs, and the metaphyseal regions of long bones, reflecting the abundance of hematopoietic red marrow in these areas [4,5,6]. Within the spine, the lumbar region is most frequently affected (52%), followed by the thoracic (36%) and cervical spine (12%) [7]. Radiation therapy (RT) is commonly employed to palliate pain caused by bone metastases, and predicting post-radiotherapy survival plays a critical role in determining appropriate treatment strategies, including a personalized medicine approach where treatment intensity can be adjusted according to individual patient prognosis [8,9].

Although overall survival (OS) after palliative radiotherapy for bone metastases has traditionally been assessed using the Cox proportional hazards (CPH) model [10,11,12], both these regression analyses and the prognostic scoring systems developed for bone metastases [10,11] share a structural assumption: each factor contributes additively, independently of the others. Whether this additive form captures the prognostic information contained in clinical variables, or whether nonlinear effects and interactions are being missed, has rarely been examined, because such an examination requires a model flexible enough to represent these structures. Against this background, the integration of artificial intelligence (AI) into this field has been increasingly reported in recent years [13]. AI encompasses a range of computational techniques, including machine learning (ML) and its advanced branch, deep learning (DL). ML, a subset of AI, involves algorithms that learn patterns from data to make predictions without explicit programming. DL, an advanced branch of ML, employs multi-layered neural networks to model complex and nonlinear relationships between input features and clinical outcomes. Compared to traditional ML methods, DL can automatically extract relevant features from raw data, reducing the need for manual feature engineering and enhancing predictive performance. This capability makes DL particularly well-suited for handling heterogeneous clinical datasets and offers the potential of superior predictive performance by capturing subtle patterns that conventional models might miss.

Despite its potential, the application of DL to survival prediction in this palliative setting remains largely unexplored, with most AI-based prognostic studies focusing on traditional ML models [14,15,16,17]. In contrast, DL has been widely applied in the realm of imaging analysis, such as for the automated detection of metastases, differentiation of lesions, and assessment of treatment response [18]. A major reason for this limited use in survival prediction is the lack of interpretability in DL models, often referred to as the “black box” problem. Recent systematic reviews or meta-analysis have shown that, although AI applications for bone metastases are increasing, DL-based prognostic models are not only scarce but also frequently lack explanatory frameworks [19,20,21].

However, this limitation of interpretability may be overcome by recent advancements in explainable AI techniques, such as Shapley Additive Explanations (SHAP) and SurvLIME [22]. By illuminating the model’s predictive rationale, explainable AIs can bridge the gap between high predictive accuracy and integration into clinical practice. Therefore, the present study aimed to develop and validate an explainable DL model to predict OS in patients receiving palliative radiotherapy for bone metastases, and to examine whether this flexible model provides additional predictive value over a standard Cox model when only routinely collected baseline variables are used.

## 2. Methods

### 2.1. Study Design and Patients

This study comprised a retrospective, single-institution cohort of patients who underwent palliative radiotherapy for bone metastases between January 2013 and August 2024. The study protocol received approval from the Institutional Review Board (approval number: 2025-010), and the requirement for informed consent was waived owing to the retrospective design of the investigation. All study procedures were conducted in accordance with relevant ethical guidelines. Clinical and dosimetric information was retrieved from the institutional radiation oncology database. All 472 eligible patients were included in the primary analysis. Patients who were alive at last contact within one year of treatment initiation (*n* = 96; median follow-up 17.5 days, interquartile range [IQR] 11–65) were treated as right-censored observations, and patients alive beyond one year were administratively censored at 365 days. To assess the influence of patients with incomplete follow-up, a sensitivity analysis restricted to the 376 patients with complete one-year follow-up was also performed.

The unit of analysis was the patient. Each patient contributed one record corresponding to the first (index) course of palliative radiotherapy for bone metastases, and the dose variable was taken from that index course. When several bone regions were irradiated within the index course, all regions are counted in Table 1, so the site counts exceed the number of patients; the irradiated site was not used as a model input.

### 2.2. Treatments

CT simulation images were acquired with patients in the supine position, immobilized according to the treated site, using an Aquilion LB large-bore scanner (Canon Medical Systems, Tochigi, Japan) with a slice thickness of 2 mm, without intravenous contrast, covering the entire target region and adjacent organs at risk. Treatment planning was performed using the Pinnacle planning system (version 9.10; Philips Medical Systems, Fitchburg, WI, USA). To enable comparison across various fractionation regimens, the prescribed dose was converted to a biologically effective dose (BED10), assuming an α/β ratio of 10. The planned prescribed dose was used as the dose variable in the primary analysis because it is fully determined at baseline. The administered dose (estimated for patients unable to complete the planned course by multiplying the prescribed dose by the proportion of fractions delivered) incorporates information that accrues after the start of follow-up and would require a landmark or time-dependent analysis; it was therefore evaluated only in a sensitivity analysis (the planned and administered doses differed in 19 of 472 patients, 4.0%). All treatments were administered using three-dimensional conformal radiotherapy (3D-CRT) with 4–10 MV photon beams produced by an Elekta Synergy linear accelerator (Elekta, Stockholm, Sweden). The predominant treatment regimen consisted of 30 Gy in 10 fractions.

### 2.3. Study Endpoints and Prognostic Grouping

The follow-up period was measured from the date of radiotherapy initiation. The primary endpoint was OS, defined as the duration from the start of treatment to death from any cause. Patients alive at last contact were censored at that date, and patients alive beyond one year were administratively censored at 365 days. In accordance with previous reports [23,24,25,26], primary tumor sites were categorized into two prognostic groups: low-risk (breast cancer, prostate cancer, and hematologic malignancies) and high-risk (all other malignancies).

### 2.4. Predictor Variables

The models used 14 baseline predictors: Eastern Cooperative Oncology Group (ECOG) performance status (grouped 0–1, 2, 3–4), age, sex, primary tumor risk group, solitary versus multiple bone metastases, number of extraosseous metastatic sites, separate indicators for brain, lung, liver, non-regional lymph node, adrenal, pleural, and peritoneal involvement, and the planned prescribed dose (BED10). Variable definitions and coding are listed in Supplementary Table S1. There were no missing values in any predictor or outcome variable. All inputs were z-standardized using the training folds only.

### 2.5. Model Development and Validation Strategy

The OS prediction model was constructed using DeepSurv, a deep learning-based Cox proportional hazards model. DeepSurv replaces the linear predictor of the Cox model with a feed-forward neural network trained by maximizing the Cox partial likelihood, thereby allowing nonlinear covariate effects and interactions while retaining the proportional-hazards framework and the ability to derive absolute survival probabilities [27]. The network consisted of two hidden layers of 32 and 16 units with ReLU activations and dropout of 0.2 (no batch normalization), trained with the Adam optimizer (learning rate 1 × 10^−3^, batch size 32, up to 300 epochs). To ensure robust evaluation, 10 repetitions of 5-fold cross-validation were performed, resulting in 50 training–testing cycles. In each fold, the dataset was randomly split into 80% for training and 20% for testing, stratified by event status. The training set was further divided into an internal training (85%) and internal validation (15%) set, and early stopping (patience = 20) was based on the internal-validation partial likelihood to prevent overfitting. The exact size of the internal validation set was adjusted by a few patients so that no training mini-batch contained fewer than four patients; a very small final mini-batch consisting only of censored patients makes the batch partial likelihood degenerate. Absolute survival probabilities were obtained from the Breslow baseline hazard estimator computed on the training data of each fold, S(t|x) = exp[−H_0_(t)·exp(g(x))], where g(x) is the network output.

### 2.6. Benchmark Models

To quantify the added value of deep learning over simpler approaches, a standard Cox proportional hazards model and a ridge-penalized Cox model (penalty selected from {0.01, 0.1, 1, 10} by inner 5-fold cross-validated partial likelihood) were fitted with the same 14 predictors on exactly the same training/test splits, and evaluated with the same metrics. Established prognostic tools such as METSSS, TEACHH, the Katagiri score, and BMETS could not be evaluated because the variables they require (e.g., laboratory values, ambulatory status) were not collected in our database.

### 2.7. Model Performance Assessment

Discrimination was quantified by the time-dependent concordance index (C-index) of Antolini et al. [28]; prediction error by the Brier score with inverse probability of censoring weighting (IPCW; Graf et al. [29]), with the censoring distribution estimated by the Kaplan–Meier method on the test set, integrated over the follow-up interval (integrated Brier score, IBS); and discrimination at fixed horizons by the IPCW cumulative/dynamic area under the curve (AUC; Uno et al. [30]) at 90, 180, and 365 days. Fold-level results are summarized as mean ± standard deviation (SD) across the 50 folds as a descriptive measure of variability; because the folds share patients and are not independent, inferential 95% CIs were instead obtained by patient-level bootstrapping (B = 1000) of the pooled out-of-fold predictions, in which each patient appears exactly once per repeat. Calibration at one year was assessed on the pooled out-of-fold predictions by comparing observed (Kaplan–Meier) and predicted mortality across deciles of predicted risk, by the observed/expected (O/E) ratio, and by the calibration slope estimated from a Cox model fitted to the complementary log-log transformed predicted risk.

### 2.8. Model Interpretability Analysis

SHAP values, with the predicted 1-year mortality risk as the model output, were computed for every patient of the held-out test set in each of the 50 folds (Monte Carlo Shapley value estimation with training-fold background samples); because each patient is in the test set exactly once per repeat, every patient contributes exactly 10 explanations to the pooled summary. Global importance is presented as mean absolute SHAP values, and local effects as a summary (beeswarm) plot. SurvLIME local importances were estimated once per patient, on the fold of the first repeat in which the patient was held out, and are summarized by the median and IQR. All analyses were performed in Python (version 3.9) using PyTorch, PyCox, scikit-learn, SHAP, and SurvLIMEpy libraries.

## 3. Results

### 3.1. Baseline Characteristics 

The baseline demographic and clinical features of the 472 patients are presented in Table 1. The median patient age was 71.4 years (range, 36–98) and male patients predominated (*n* = 291, 61.7%). Regarding ECOG PS, 175 patients (37.1%) had PS 0–1, 117 (24.8%) had PS 2, and 180 (38.1%) had PS 3–4. Lung cancer was the most common primary site (*n* = 211, 44.7%), followed by breast (*n* = 66, 14.0%), prostate (*n* = 54, 11.4%), and colorectal cancer (*n* = 38, 8.1%). Tumors classified as low-risk malignancies (breast, prostate, and hematologic) were identified in 126 patients (26.7%), whereas the remaining 346 patients (73.3%) had high-risk malignancies. Multiple bone metastases were observed in 380 patients (80.5%). The distribution of extraosseous metastatic sites was: none in 195 patients (41.3%), one site in 137 (29.0%), and two or more sites in 140 (29.7%). The lung was involved in 118 patients (25.0%), the liver in 121 (25.6%), and non-regional lymph nodes in 95 (20.1%). The vertebral column was the most commonly irradiated region (*n* = 258, 54.7%), followed by the pelvis (*n* = 135, 28.6%). Regarding the planned dose (BED10), 39 Gy was most frequent (*n* = 313, 66.3%), followed by >39 Gy (*n* = 99, 21.0%) and <39 Gy (*n* = 60, 12.7%).

### 3.2. Survival Outcomes

**Figure 1:**
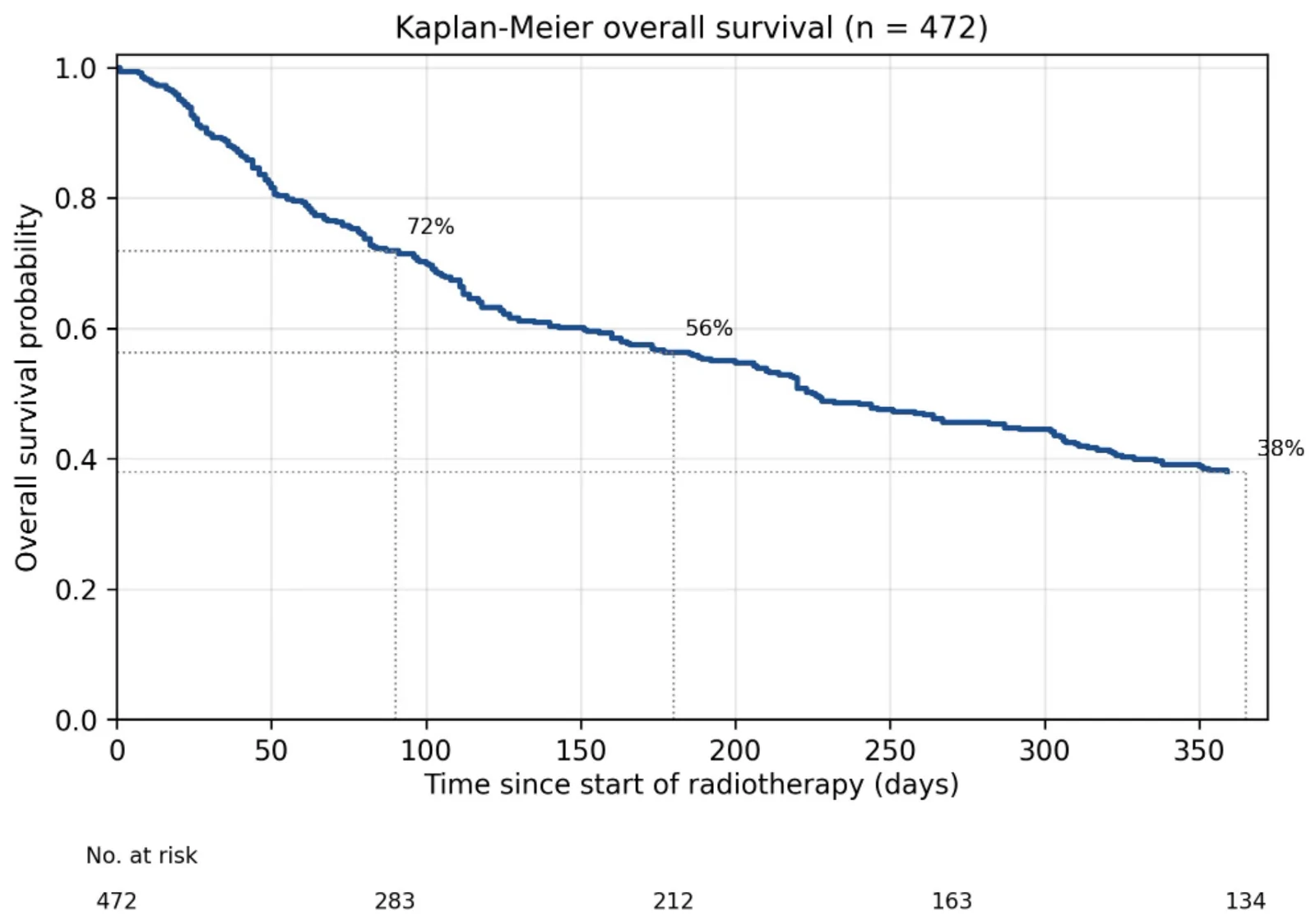
Kaplan–Meier overall survival of the full cohort (*n* = 472) Kaplan–Meier estimate of overall survival from the start of palliative radiotherapy, administratively censored at 365 days, with numbers at risk. Median OS was 225 days (95% CI: 189–287); OS was 71.9% at 3 months, 56.4% at 6 months, and 38.0% at 1 year. OS, overall survival; CI, confidence interval.

Within one year of treatment initiation, 242 patients (51.3%) died. The median OS was 225 days (approximately 7.4 months; 95% CI: 189–287), and Kaplan–Meier OS estimates were 71.9% (95% CI: 67.5–75.9) at 3 months, 56.4% (95% CI: 50.8–61.0) at 6 months, and 38.0% (95% CI: 33.3–43.0) at 1 year (Figure 1). A total of 134 patients were alive with at least 365 days of follow-up; the remaining 96 patients were censored earlier (median follow-up 17.5 days, IQR 11–65).

### 3.3. Cross-Validation Performance, Model Comparison, and Calibration

**Figure 2:**
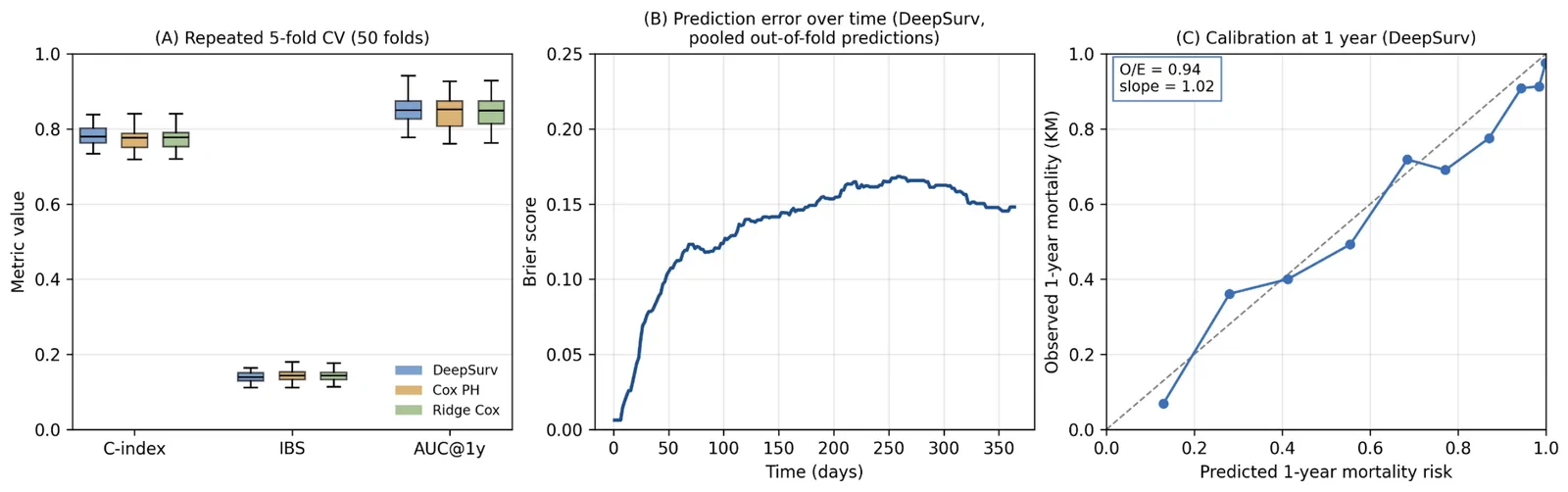
Cross-validation performance, prediction error, and calibration of the survival models. (**A**) Distribution of the time-dependent C-index, IBS, and 1-year AUC across the 50 folds of repeated five-fold cross-validation (K = 5, R = 10) for DeepSurv, the standard Cox model, and the ridge-penalized Cox model. (**B**) Prediction error (Brier score) over time for DeepSurv, computed on the pooled out-of-fold predictions. (**C**) Calibration of the pooled out-of-fold DeepSurv predictions at one year: observed (Kaplan–Meier) versus predicted 1-year mortality across deciles of predicted risk, with the observed/expected ratio and calibration slope. OS, overall survival; C-index, concordance index; CI, confidence interval; IBS, integrated Brier score; AUC, area under the curve; SD, standard deviation.

Figure 2A shows the distribution of performance metrics across the 50 folds for DeepSurv and the two Cox benchmarks, and Table 2 summarizes the pooled out-of-fold performance with bootstrap CIs. The DeepSurv model achieved a pooled time-dependent C-index of 0.779 (95% CI: 0.751–0.807; fold-level mean ± SD 0.781 ± 0.026), an IBS of 0.135 (95% CI: 0.122–0.149), and AUCs of 0.892 (95% CI: 0.857–0.925), 0.862 (95% CI: 0.822–0.895), and 0.856 (95% CI: 0.814–0.895) at 90, 180, and 365 days, respectively. Discrimination was comparable to the standard Cox model (pooled C-index 0.763, 95% CI: 0.737–0.789) and the ridge-penalized Cox model (0.763, 95% CI: 0.739–0.792). Figure 2B depicts the prediction error (Brier score) over time, computed on the pooled out-of-fold predictions. Figure 2C shows the calibration of the pooled out-of-fold predictions at one year: observed and predicted mortality agreed across risk deciles, with an O/E ratio of 0.94 and a calibration slope of 1.02 (Cox model: O/E 0.99, slope 0.90).

### 3.4. Multivariable Cox Analysis

In the multivariable Cox model of the full cohort (Table 3), poorer performance status (hazard ratio [HR] 2.21 per SD, 95% CI: 1.89–2.57, *p* < 0.001), high-risk primary tumor type (HR 1.33, 95% CI: 1.14–1.55, *p* < 0.001), liver metastases (HR 1.28, 95% CI: 1.12–1.46, *p* < 0.001), multiple bone metastases (HR 1.28, 95% CI: 1.09–1.50, *p* = 0.003), non-regional lymph node metastases (HR 1.22, 95% CI: 1.07–1.39, *p* = 0.002), and lung metastases (HR 1.17, 95% CI: 1.02–1.33, *p* = 0.021) were independently associated with worse OS, whereas a higher planned dose was associated with better OS (HR 0.71 per SD, 95% CI: 0.60–0.84, *p* < 0.001). Male sex showed a borderline association (HR 1.15, 95% CI: 1.00–1.32, *p* = 0.055).

### 3.5. SHAP Analysis of the DeepSurv Model

**Figure 3:**
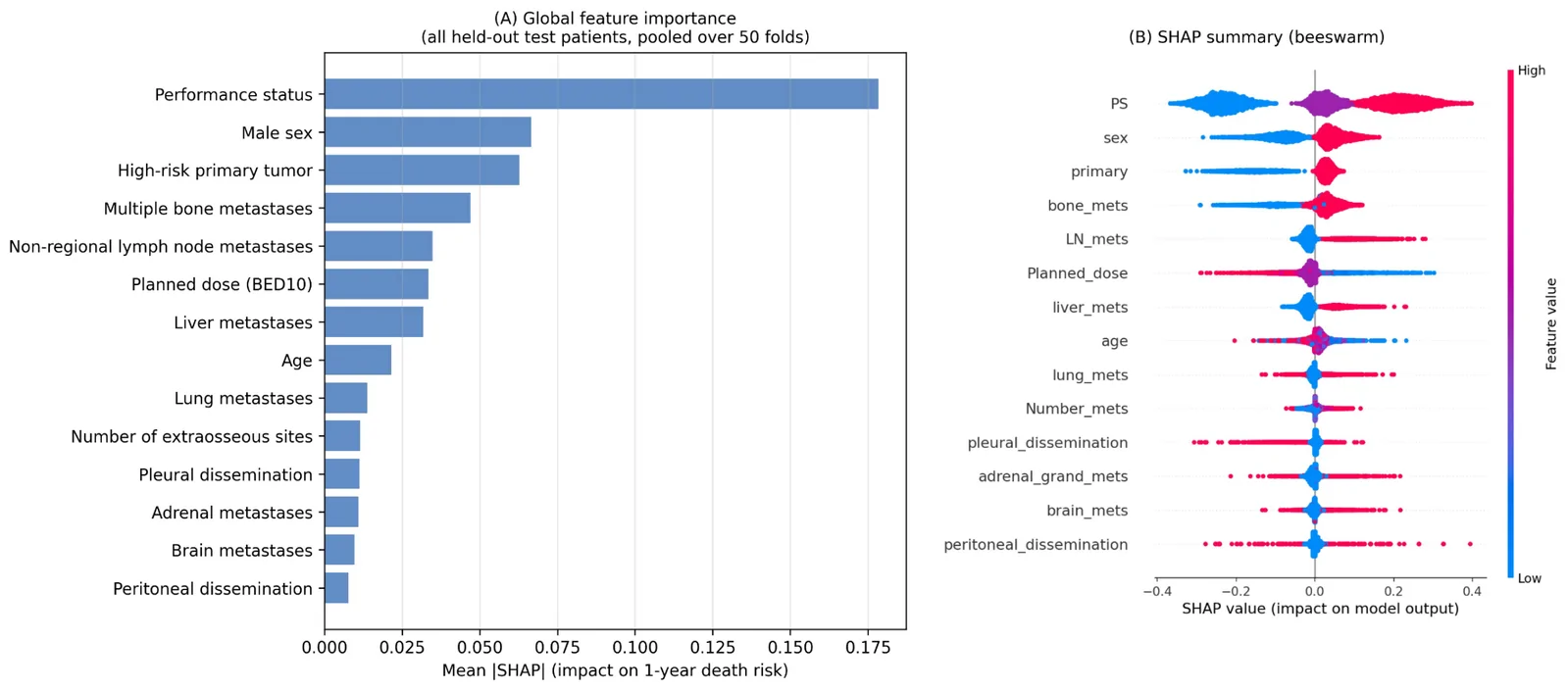
Global and local feature importance based on SHAP analysis of the DeepSurv model (**A**) Global feature importance ranked by mean absolute SHAP values, computed for every patient of the held-out test sets and pooled across 50 cross-validation folds. The top predictors were PS (0.178), male sex (0.067), high-risk primary tumor type (0.063), multiple bone metastases (0.047), non-regional lymph node metastases (0.035), planned dose (0.033), and liver metastases (0.032). (**B**) Summary (beeswarm) plot of local feature effects on the predicted 1-year death risk; color indicates the feature value (red = high, blue = low) and horizontal position the impact on mortality (positive = higher predicted mortality); variable labels in panel (**B**) correspond to the coding listed in Supplementary Table S1. SHAP, Shapley additive explanations; PS, performance status; OS, overall survival.

Figure 3A shows the global feature importance ranked by mean absolute SHAP values, computed on the held-out test sets and pooled across all 50 folds. PS made by far the greatest contribution (mean |SHAP| 0.178), followed by male sex (0.067), high-risk primary tumor type (0.063), multiple bone metastases (0.047), non-regional lymph node metastases (0.035), planned dose (0.033), and liver metastases (0.032). Figure 3B presents the summary (beeswarm) plot of local feature effects on the predicted 1-year mortality risk. Poorer PS, male sex, high-risk primary tumor type, multiple bone metastases, and nodal involvement contributed to a higher predicted risk, whereas a higher planned dose was the only leading feature associated with a lower predicted risk.

### 3.6. SurvLIME Analysis of the DeepSurv Model

**Figure 4:**
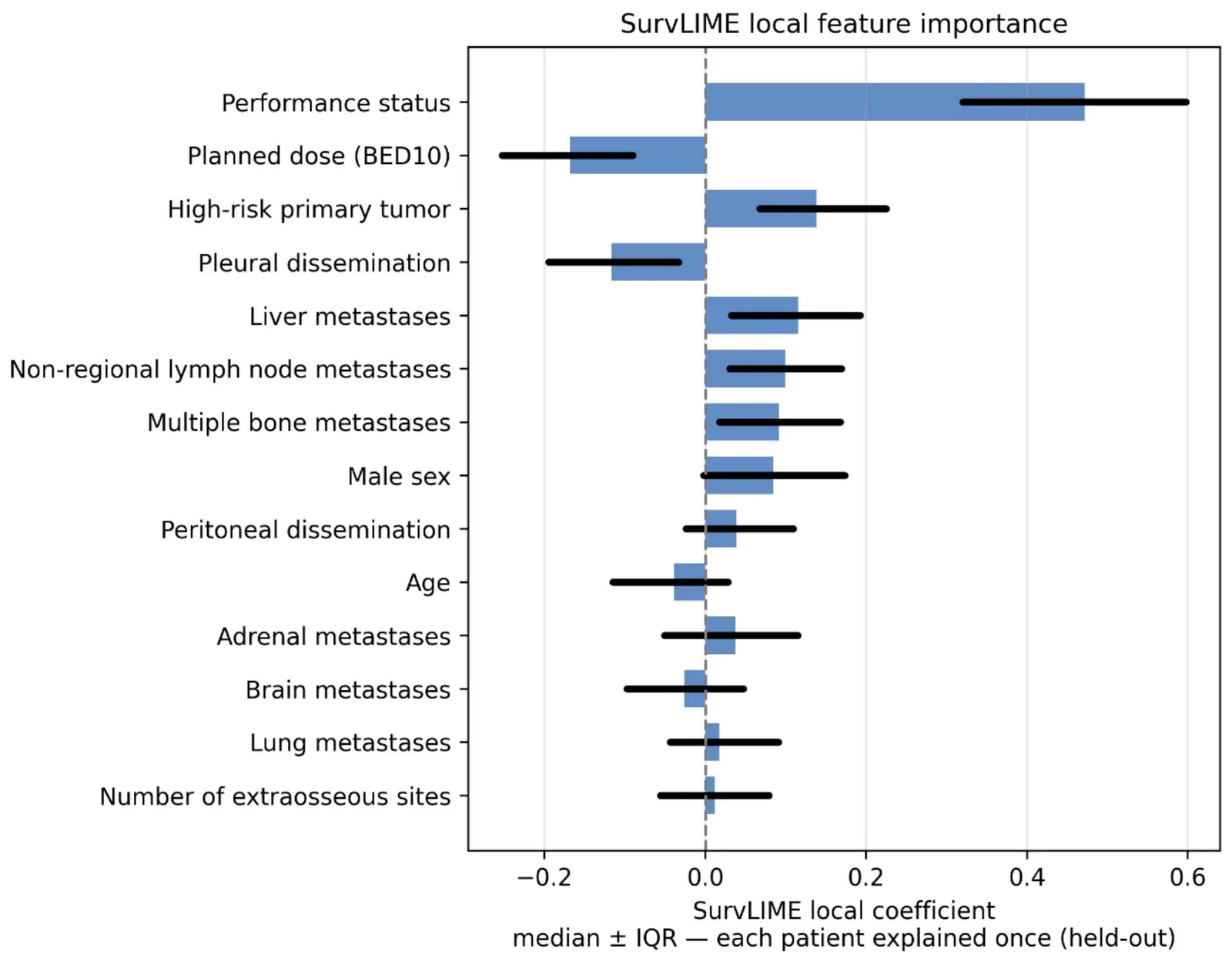
Local feature importance based on SurvLIME analysis of the DeepSurv model Median SurvLIME coefficients with IQR (bars and whiskers), estimated once per patient on held-out data. Positive coefficients are associated with higher 1-year mortality. The top contributing features were poor PS (+0.472 [+0.320 to +0.598]), planned dose (−0.168 [−0.253 to −0.091]), high-risk primary tumor type (+0.138 [+0.068 to +0.225]), liver metastases (+0.116 [+0.033 to +0.193]), and non-regional lymph node metastases (+0.100 [+0.030 to +0.169]). OS, overall survival; PS, performance status; IQR, interquartile range.

Figure 4 shows the SurvLIME local feature importances, estimated once per patient on held-out data. Positive coefficients indicate a contribution toward higher 1-year mortality. The top contributing features were poor PS (median [IQR]: +0.472 [+0.320 to +0.598]), planned dose (−0.168 [−0.253 to −0.091]), high-risk primary tumor type (+0.138 [+0.068 to +0.225]), liver metastases (+0.116 [+0.033 to +0.193]), and non-regional lymph node metastases (+0.100 [+0.030 to +0.169]); the planned dose again showed a consistent contribution toward lower predicted mortality.

### 3.7. Sensitivity Analyses

Restricting the analysis to the 376 patients with complete one-year follow-up gave consistent results (fold-level C-index 0.773 ± 0.025, IBS 0.143 ± 0.015, 1-year AUC 0.847 ± 0.038). Using the administered dose instead of the planned dose also gave nearly identical results (C-index 0.793 ± 0.025, IBS 0.136 ± 0.014, 1-year AUC 0.857 ± 0.038). Within prognostic subgroups, discrimination and calibration of the pooled out-of-fold predictions were preserved (high-risk primaries: C-index 0.746, O/E 0.95; low-risk primaries: C-index 0.832, O/E 0.94).

## 4. Discussion

This study developed and evaluated an explainable DL model to predict OS following palliative radiotherapy for bone metastases. The main findings of this study are summarized as follows: (1) the explainable DL model predicted 1-year OS with a pooled C-index of 0.78 and an observed/expected ratio of 0.94; (2) its performance was comparable to, though not decisively better than, standard and penalized Cox models fitted on identical data splits; and (3) the explainability analyses consistently identified performance status as the dominant predictor, with the planned dose contributing a smaller, negatively directed (protective) association that must be interpreted with caution.

Within the AI domain, prognostic modeling of OS after palliative radiotherapy for bone metastases has predominantly relied on ML-based models rather than DL approaches [19,20,21]. Cilla et al. developed an explainable ML model (using an extreme gradient boosting algorithm) to predict 1-year OS, achieving high accuracy (AUC = 0.805) while identifying key prognostic factors such as interleukin-8, hemoglobin, and lymphocyte count [14]. Elledge et al. externally validated the Bone Metastases Ensemble Trees for Survival (BMETS) ML model for predicting survival after palliative radiotherapy for bone metastases, demonstrating consistent accuracy and generalizability [16]. Nieder et al. independently validated the BMETS machine learning model in a Norwegian cohort. While this validation confirmed its prognostic utility, it also revealed limitations such as a tendency to overpredict survival: 59% of patients had an actual survival shorter than predicted, and 16% of those who died within 3 months had been assigned a predicted median survival exceeding 6 months [17].

A barrier to the widespread adoption of DL for survival prediction has been its lack of interpretability. However, emerging explainable AI techniques offer a promising path to overcome this limitation by elucidating the model’s decision-making process. While explainable DL models are beginning to appear, as exemplified by the work of Astley et al. on survival prediction for non-small-cell lung cancer patients undergoing radical radiotherapy [22], their application in the context of palliative radiotherapy for bone metastases remains scarce. In the present study, we developed an explainable DL model using DeepSurv, which demonstrated robust predictive performance in this specific palliative cohort. The discriminative performance of DeepSurv was comparable to that of a well-specified Cox model: in this moderately sized cohort with 14 tabular predictors, the flexibility of the neural network provided no measurable advantage over the linear model. Because the network can represent nonlinear effects and interactions without prespecification, this equivalence, together with the agreement between the SHAP rankings and the Cox coefficients (Table 3), suggests that the prognostic information contained in these baseline variables is essentially additive and log-linear; the flexible model thus examined an assumption that the Cox model itself can only impose. This bears on existing practice: the prognostic scores in routine use for bone metastases are additive point systems [23,24,25,26] whose adequacy has been assumed rather than tested. Our analysis supports the additive form for the clinical factors that constitute the core of these scores, namely performance status, primary tumor type, and the extent of metastatic disease; whether additivity also holds for score components we could not evaluate, such as laboratory values, remains untested. The contribution of the present work therefore lies less in a performance advantage than in a complete, explainable deep learning survival pipeline with patient-level uncertainty quantification, calibration assessment, and individual-level explanation on held-out data. In a field in which DL prognostic models are increasingly reported [19,20,21], such direct comparisons against simpler models help direct modeling effort toward settings in which flexibility can pay off; the same pipeline can be reused when richer inputs, such as imaging, laboratory trajectories, or dosimetric data [31], become available.

Furthermore, the explainable AI techniques applied in this study, SHAP and SurvLIME, allowed the quantification and visualization of the relative contribution of each clinical and dosimetric factor to OS prediction. SHAP provides global interpretability by estimating the overall importance and directional impact of each feature, whereas SurvLIME offers local interpretability by explaining feature contributions for individual predictions. The combined use of these complementary methods enables a more comprehensive understanding of the model’s behavior. Both methods identified poor PS as by far the strongest predictor, followed by male sex, high-risk primary tumor type, and the burden of metastatic disease; the planned dose contributed a smaller association, and was the only leading feature whose higher values were associated with lower predicted mortality. The multivariable Cox analysis was consistent with this pattern (Table 3).

PS is well-established as a robust prognostic factor regardless of bone metastasis status, across a wide range of cancer types [23,24,32,33,34,35,36,37,38,39]. Similarly, the stratification of primary tumor types into risk categories was based on established results from prognostic models for patients with bone metastases [23,24,25,26]. Beyond these well-defined patient and tumor characteristics, the prognostic contribution of radiotherapy-specific variables has been less clear [40]. The optimal fractionation schedule for palliative radiotherapy in bone metastases has been a subject of longstanding debate [41], and randomized trials have shown similar palliative outcomes for short-course (e.g., 8 Gy in 1 fraction) and long-course (e.g., 30 Gy in 10 fractions) regimens [33,42]. Although several observational reports have suggested an association between higher radiation doses and longer survival [43,44,45], such associations are highly susceptible to confounding by indication.

The association between a higher planned dose and longer survival observed in this study must not be interpreted as evidence of a treatment benefit. In routine practice, longer or higher-dose fractionation schedules are preferentially prescribed to patients judged to have a better prognosis, whereas single-fraction or short-course regimens are chosen for patients with limited life expectancy. The dose variable therefore partly encodes the treating physician’s clinical judgment, and its predictive contribution most likely reflects confounding by indication. SHAP and SurvLIME quantify how the model uses a variable for prediction; they do not establish causality. Although the dose association persisted after adjustment for the measured clinical factors in the multivariable Cox model, residual confounding by unmeasured factors (e.g., laboratory values, planned systemic therapy) cannot be excluded. Accordingly, our findings support the use of the prescribed regimen as a prognostic feature, but they provide no evidence that escalating the dose would improve survival; only prospective randomized comparisons could address that question.

All patients in this cohort were treated with conventional 3D-CRT. Stereotactic body radiotherapy (SBRT) is increasingly used for patients with a limited number of bone metastases. Prognostic models for this oligometastatic population, in which expected survival, treatment intent, and dose–fractionation considerations differ from those of conventional palliation, would require dedicated development; our model was trained exclusively on patients treated with 3D-CRT and should not be applied to patients treated with SBRT without retraining and validation.

### 4.1. Clinical Implications

The explainable nature of this model is crucial, as it has the potential to assist clinicians in estimating individualized survival probabilities. This, in turn, can support evidence-based decision-making regarding treatment goals and fractionation schedules. By allowing clinicians to ascertain the key prognostic contributors, the model’s interpretability may enhance clinician confidence and facilitate the integration of such AI-assisted prognostic tools into routine radiation oncology practice. The adequacy of an additive model also has a practical consequence: the prognostic information of these baseline variables can be delivered at the bedside as a simple score or nomogram, without dedicated AI infrastructure, and without loss of predictive performance. The time-dependent AUCs at 90 and 180 days (0.892 and 0.862) are of particular clinical interest, because expected survival at these horizons is central to the choice between single-fraction and multi-fraction palliation. The dose–survival association identified by the explainability analyses describes how the model uses the prescribed regimen as a prognostic signal; it is not a rationale for dose escalation.

### 4.2. Limitation

First, the retrospective, single-institution design means that treatment selection, in particular the choice of fractionation schedule, was driven by clinical judgment; the dose–survival association is therefore likely to be affected by confounding by indication, and unmeasured confounders (e.g., laboratory values, systemic therapy after radiotherapy) cannot be excluded. Second, the case mix of the cohort, dominated by lung cancer and other high-risk primaries, reflects a single-institution referral pattern; although discrimination and calibration were preserved within both prognostic subgroups (C-index 0.746 and O/E 0.95 in high-risk, 0.832 and 0.94 in low-risk primaries), no external validation was performed, internally validated performance is typically optimistic relative to application in new institutions, and the findings should be regarded as hypothesis-generating until externally validated. Third, established prognostic scores such as METSSS [10], TEACHH [46], the Katagiri score [23], and BMETS [16] could not be compared head-to-head because the variables they require (e.g., laboratory data, ambulatory status) were not available in our database; for the same reason, the additivity finding applies to the measured clinical variables and not to these unmeasured score components. Fourth, all patients were treated with 3D-CRT, and the results may not extend to SBRT. Fifth, primary tumors were classified into two risk categories according to previous literature [23,24,25,26]; however, other prognostic classification frameworks have also been developed [46,47,48]. Finally, the cohort remains moderate in size, and a single network architecture was evaluated; weak nonlinear effects or interactions below the detection limit of this sample cannot be excluded, which is why the additive structure is described as suggested rather than established. Prospective multicenter evaluation would be required to determine whether the model retains its discrimination and calibration in other institutions.

## 5. Conclusions

An explainable DL model to predict OS following palliative radiotherapy for bone metastases was developed and internally validated; its discrimination and calibration were comparable to those of a well-specified Cox model, and its feature attributions agreed with the Cox coefficients, suggesting an essentially additive prognostic structure whose information can be delivered at the bedside as a simple score or nomogram, without dedicated AI infrastructure and without loss of predictive performance. The combined use of SHAP and SurvLIME made the model’s predictions transparent at the cohort and individual levels and identified established clinical factors, most prominently performance status, as the dominant predictors. Pending external validation, such explainable survival prediction may support individualized decisions regarding treatment goals and fractionation schedules.

## Figures and Tables

**Table 1:** Patient Characteristics.

Characteristics	No. of Patients (%) (*n* = 472)
Age	
<71	224 (47.5)
≥71	248 (52.5)
Sex	
Male	291 (61.7)
Female	181 (38.3)
ECOG PS	
0–1	175 (37.1)
2	117 (24.8)
3–4	180 (38.1)
Primary tumor sites	
Lung	211 (44.7)
Breast	66 (14.0)
Prostate	54 (11.4)
Colorectal	38 (8.1)
Kidney	18 (3.8)
Esophagus/gastric	16 (3.4)
Renal pelvis/ureter	12 (2.5)
Liver (HCC)	11 (2.3)
Oral/pharyngeal	10 (2.1)
Bladder	8 (1.7)
Pancreatic/hepatobiliary	7 (1.5)
Hematologic malignancy	6 (1.3)
Uterus	5 (1.1)
Cancer of unknown primary	4 (0.8)
Ovary	3 (0.6)
Pulmonary artery sarcoma	2 (0.4)
Pleura (malignant pleural mesothelioma)	1 (0.2)
Risk of primary tumor	
High-risk group	346 (73.3)
Low-risk group	126 (26.7)
Number of bone metastases	
1	92 (19.5)
≥2	380 (80.5)
Number of extraosseous distant metastases	
0	195 (41.3)
1	137 (29.0)
≥2	140 (29.7)
Site of distant metastases	
Brain	61 (12.9)
Lung	118 (25.0)
Liver	121 (25.6)
Adrenal gland	41 (8.7)
Non-regional lymph nodes	95 (20.1)
Pleural dissemination	56 (11.9)
Peritoneal dissemination	16 (3.4)
Others	86 (18.2)
Sites of palliative RT for bone metastases	
Vertebral	258 (54.7)
Pelvis	135 (28.6)
Extremity (e.g., femur, humerus)	46 (9.7)
Rib/Sternum	46 (9.7)
Skull	11 (2.3)
Others	20 (4.2)
Planned dose (BED10)	
<39.0 Gy	60 (12.7)
39.0 Gy	313 (66.3)
>39.0 Gy	99 (21.0)

BED10, biologically effective dose with α/β = 10; ECOG PS, Eastern Cooperative Oncology Group Performance Status; RT, radiation therapy. Site counts enumerate all bone regions treated within the index course, so they may exceed the number of patients.

**Table 2:** Predictive performance of DeepSurv and the Cox benchmarks (primary analysis, *n* = 472).

	DeepSurv	Cox PH	Ridge Cox
C-index, fold mean ± SD	0.781 ± 0.026	0.771 ± 0.026	0.772 ± 0.026
C-index, pooled (95% CI)	0.779 (0.751–0.807)	0.763 (0.737–0.789)	0.763 (0.739–0.792)
IBS, fold mean ± SD	0.140 ± 0.014	0.143 ± 0.014	0.143 ± 0.013
IBS, pooled (95% CI)	0.135 (0.122–0.149)	0.142 (0.128–0.156)	0.142 (0.130–0.154)
AUC at 90 d, pooled (95% CI)	0.892 (0.857–0.925)	0.879 (0.839–0.913)	0.877 (0.838–0.914)
AUC at 180 d, pooled (95% CI)	0.862 (0.822–0.895)	0.847 (0.806–0.885)	0.847 (0.807–0.887)
AUC at 365 d, pooled (95% CI)	0.856 (0.814–0.895)	0.841 (0.797–0.880)	0.843 (0.803–0.881)

Fold-level values are mean ± SD across the 50 folds (descriptive). Pooled values are computed on the pooled out-of-fold predictions with 95% CIs from patient-level bootstrapping (B = 1000). C-index, time-dependent concordance index (Antolini); IBS, integrated Brier score (Graf); AUC, cumulative/dynamic area under the curve (Uno); IPCW, inverse probability of censoring weighting; CI, confidence interval; SD, standard deviation.

**Table 3:** Multivariable Cox proportional hazards analysis of overall survival (*n* = 472).

Variable	HR per SD	95% CI	*p*
Performance status (0–1/2/3–4)	2.21	1.89–2.57	<0.001
Planned dose (BED10)	0.71	0.60–0.84	<0.001
High-risk primary tumor	1.33	1.14–1.55	<0.001
Liver metastases	1.28	1.12–1.46	<0.001
Multiple bone metastases	1.28	1.09–1.50	0.003
Non-regional lymph node metastases	1.22	1.07–1.39	0.002
Lung metastases	1.17	1.02–1.33	0.021
Male sex	1.15	1.00–1.32	0.055
Peritoneal dissemination	1.09	0.95–1.25	0.211
Adrenal metastases	1.09	0.96–1.22	0.179
Age	1.03	0.90–1.19	0.652
Pleural dissemination	0.90	0.80–1.02	0.105
Brain metastases	0.90	0.79–1.02	0.101

Hazard ratios are expressed per standard deviation of each (standardized) predictor. The number of extraosseous metastatic sites was excluded from this table because it is the exact sum of the seven site indicators (exact collinearity); it was retained in the prediction models, whose fitted values are unaffected. HR, hazard ratio; CI, confidence interval; SD, standard deviation; BED10, biologically effective dose with α/β = 10.

## Data Availability

The complete analysis code (model training, evaluation, and explainability pipeline) is provided as Supplementary Materials. The de-identified patient-level dataset supporting the findings of this study is available from the corresponding author upon reasonable request, subject to institutional review board requirements.

## Supplementary Materials

The following supporting information can be downloaded at: https://www.mdpi.com/article/10.3390/curroncol33090528/s1, Table S1: Predictor variables and coding; File S1: Complete analysis code for model training, evaluation, and explainability analyses.

## Abbreviations

3D-CRT	three-dimensional conformal radiotherapy
AI	artificial intelligence
AUC	area under the curve
BED10	biologically effective dose with α/β = 10
CI	confidence interval
C-index	concordance index
CPH	cox proportional hazards
DL	deep learning
ECOG PS	Eastern Cooperative Oncology Group Performance Status
HR	hazard ratio
IBS	integrated Brier score
IPCW	inverse probability of censoring weighting
IQR	interquartile range
KM	Kaplan–Meier
ML	machine learning
O/E	observed/expected ratio
OAR	organs at risk
OS	overall survival
PS	performance status
RT	radiation therapy
SBRT	stereotactic body radiotherapy
SHAP	shapley additive explanations

## References

[1] Nystrom J.S., Weiner J.M., Heffelfinger-Juttner J., Irwin L.E., Bateman J.R., Wolf R.M. (1977). Metastatic and histologic presentations in unknown primary cancer. Semin. Oncol..

[2] Coleman R.E. (2001). Metastatic bone disease: Clinical features, pathophysiology and treatment strategies. Cancer Treat. Rev..

[3] Greco T., Fulchignoni C., Cianni L., Maccauro G., Perisano C. (2022). Surgical management of tibial metastases: A systematic review. Acta Biomed..

[4] Bussard K.M., Gay C.V., Mastro A.M. (2008). The bone microenvironment in metastasis; what is special about bone?. Cancer Metastasis Rev..

[5] Roberts C.C., Daffner R.H., Weissman B.N., Bancroft L., Bennett D.L., Blebea J.S., Bruno M.A., Fries I.B., Germano I.M., Holly L. (2010). ACR appropriateness criteria^®^ on metastatic bone disease. J. Am. Coll. Radiol..

[6] Saha S., Burke C., Desai A., Vijayanathan S., Gnanasegaran G. (2013). SPECT-CT: Applications in musculoskeletal radiology. Br. J. Radiol..

[7] Talbot J.N., Paycha F., Balogova S. (2011). Diagnosis of bone metastasis: Recent comparative studies of imaging modalities. Q. J. Nucl. Med. Mol. Imaging.

[8] Watanabe Y., Tomoda T., Iwata A., Matsuno H., Hayakawa H., Nagata T. (2026). A novel scoring system based on post-radiotherapy changes in pain, performance status, and adverse events to stratify survival in bone metastases. Sci. Rep..

[9] Watanabe Y., Nakamura S., Ichikawa Y., Ii N., Kawamura T., Kondo E., Ikeda T., Nomoto Y., Sakuma H. (2021). Early alteration in apparent diffusion coefficient and tumor volume in cervical cancer treated with chemoradiotherapy or radiotherapy: Incremental prognostic value over pretreatment assessments. Radiother. Oncol..

[10] Zaorsky N.G., Liang M., Patel R., Lin C., Tchelebi L.T., Newport K.B., Fox E.J., Wang M. (2021). Survival after palliative radiation therapy for cancer: The METSSS model. Radiother. Oncol..

[11] Sakurai T., Takamatsu S., Shimoyachi N., Shibata S., Makino M., Ohashi S., Taima Y., Minamikawa R., Kumano T., Gabata T. (2022). Prediction of post-radiotherapy survival for bone metastases: A comparison of the 3-variable number of risk factors model with the new Katagiri scoring system. J. Radiat. Res..

[12] Schmid R.K., Johnstone C.A., Robbins J.R. (2022). Palliative radiation for bone metastases from hepatocellular carcinoma: Practice patterns and the amount of remaining life spent receiving treatment. Ann. Palliat. Med..

[13] Papalia G.F., Brigato P., Sisca L., Maltese G., Faiella E., Santucci D., Pantano F., Vincenzi B., Tonini G., Papalia R. (2024). Artificial intelligence in detection, management, and prognosis of bone metastasis: A systematic review. Cancers.

[14] Cilla S., Rossi R., Habberstad R., Klepstad P., Dall’Agata M., Kaasa S., Valenti V., Donati C.M., Maltoni M., Morganti A.G. (2024). Explainable machine learning model to predict overall survival in patients treated with palliative radiotherapy for bone metastases. JCO Clin. Cancer Inform..

[15] Yen H.K., Hu M.H., Zijlstra H., Groot O.Q., Hsieh H.C., Yang J.J., Karhade A.V., Chen P.-C., Huang P.-H., Chen Y.-H. (2022). Prognostic significance of lab data and performance comparison by validating survival prediction models for patients with spinal metastases after radiotherapy. Radiother. Oncol..

[16] Elledge C.R., LaVigne A.W., Fiksel J., Wright J.L., McNutt T., Kleinberg L.R., Hu C., Smith T.J., Zeger S., DeWeese T.L. (2021). External validation of the bone metastases ensemble trees for survival (BMETS) machine learning model to predict survival in patients with symptomatic bone metastases. JCO Clin. Cancer Inform..

[17] Nieder C., Mannsåker B., Yobuta R. (2021). Independent validation of a comprehensive machine learning approach predicting survival after radiotherapy for bone metastases. Anticancer Res..

[18] Ong W., Lee A., Tan W.C., Fong K.T., Lai D.D., Tan Y.L., Low X.Z., Ge S., Makmur A., Ong S.J. (2024). Oncologic applications of artificial intelligence and deep learning methods in CT spine imaging—A systematic review. Cancers.

[19] van der Linden L.R., Vavliakis I., de Groot T.M., Jutte P.C., Doornberg J.N., Lozano-Calderon S.A., Groot O.Q. (2025). Artificial Intelligence in bone Metastases: A systematic review in guideline adherence of 92 studies. J. Bone Oncol..

[20] Wilson S.B., Ward J., Munjal V., Lam C.S., Patel M., Zhang P., Xu D.S., Chakravarthy V.B. (2025). Machine learning in spine oncology: A narrative review. Glob. Spine J..

[21] Sanker V., Dawer P., Thaller A., Li Z., Heesen P., Hariharan S., Nordin E.O.R., Cavagnaro M.J., Ratliff J., Desai A. (2025). Artificial Intelligence Models for Predicting Outcomes in Spinal Metastasis: A Systematic Review and Meta-Analysis. J. Clin. Med..

[22] Astley J.R., Reilly J.M., Robinson S., Wild J.M., Hatton M.Q., Tahir B.A. (2024). Explainable deep learning-based survival prediction for non-small cell lung cancer patients undergoing radical radiotherapy. Radiother. Oncol..

[23] Katagiri H., Okada R., Takagi T., Takahashi M., Murata H., Harada H., Nishimura T., Asakura H., Ogawa H. (2014). New prognostic factors and scoring system for patients with skeletal metastasis. Cancer Med..

[24] Mizumoto M., Harada H., Asakura H., Hashimoto T., Furutani K., Hashii H., Takagi T., Katagiri H., Takahashi M., Nishimura T. (2008). Prognostic factors and a scoring system for survival after radiotherapy for metastases to the spinal column: A review of 544 patients at Shizuoka Cancer Center Hospital. Cancer.

[25] Kubota H., Soejima T., Sulaiman N.S., Sekii S., Matsumoto Y., Ota Y., Tsujino K., Fujita I., Fujimoto T., Morishita M. (2019). Predicting the survival of patients with bone metastases treated with radiation therapy: A validation study of the Katagiri scoring system. Radiat. Oncol..

[26] Takeda K., Umezawa R., Yamamoto T., Takahashi N., Suzuki Y., Kishida K., Omata S., Jingu K. (2023). Survival prediction nomogram for patients with vertebral bone metastases treated with palliative radiotherapy. Rep. Pract. Oncol. Radiother..

[27] Katzman J.L., Shaham U., Cloninger A., Bates J., Jiang T., Kluger Y. (2018). DeepSurv: Personalized treatment recommender system using a Cox proportional hazards deep neural network. BMC Med. Res. Methodol..

[28] Antolini L., Boracchi P., Biganzoli E. (2005). A time-dependent discrimination index for survival data. Stat. Med..

[29] Graf E., Schmoor C., Sauerbrei W., Schumacher M. (1999). Assessment and comparison of prognostic classification schemes for survival data. Stat. Med..

[30] Uno H., Cai T., Tian L., Wei L.J. (2007). Evaluating prediction rules for t-year survivors with censored regression models. J. Am. Stat. Assoc..

[31] Appelt A.L., Elhaminia B., Gooya A., Gilbert A., Nix M. (2022). Deep learning for radiotherapy outcome prediction using dose data—A review. Clin. Oncol..

[32] Tam A., Scarpi E., Maltoni M.C., Rossi R., Fairchild A., Dennis K., Vaska M., Kerba M. (2024). A Systematic Review of Prognostic Factors in Patients with Cancer Receiving Palliative Radiotherapy: Evidence-Based Recommendations. Cancers.

[33] van der Linden Y.M., Dijkstra S.P., Vonk E.J., Marijnen C.A., Leer J.W. (2005). Dutch Bone Metastasis Study Group. Prediction of survival in patients with metastases in the spinal column: Results based on a randomized trial of radiotherapy. Cancer.

[34] Westhoff P.G., de Graeff A., Monninkhof E.M., Bollen L., Dijkstra S.P., van der Steen-Banasik E.M., van Vulpen M., Leer J.W.H., Marijnen C.A., van der Linden Y.M. (2014). An easy tool to predict survival in patients receiving radiation therapy for painful bone metastases. Int. J. Radiat. Oncol. Biol. Phys..

[35] Watanabe Y., Koide Y., Shimizu H., Aoyama T., Shindo Y., Hashimoto S., Tachibana H., Kodaira T. (2024). Risk stratification by combination of heart and lung dose in locally advanced non-small-cell lung cancer after radiotherapy. Cancers.

[36] Watanabe Y., Koide Y., Shimizu H., Aoyama T., Shindo Y., Hashimoto S., Tachibana H., Kodaira T. (2025). Combined Impact of Coronary Artery Calcification and Heart Radiation Dose on Overall Survival in Locally Advanced Non-Small Cell Lung Cancer: Who Benefits Most from Reducing Heart Radiation Dose?. Clin. Lung Cancer.

[37] Lehtonen M., Heiskanen L., Reinikainen P., Kellokumpu-Lehtinen P.L. (2020). Both comorbidity and worse performance status are associated with poorer overall survival after external beam radiotherapy for prostate cancer. BMC Cancer.

[38] Song T., Wan Q., Yu W., Li J., Lu S., Xie C., Wang H., Fang M. (2017). Pretreatment nutritional risk scores and performance status are prognostic factors in esophageal cancer patients treated with definitive chemoradiotherapy. Oncotarget.

[39] Watanabe Y., Koide Y., Aoyama T., Hashimoto S., Tachibana H., Kodaira T. (2025). The prognostic effect of coronary artery calcification in locally advanced non-small cell lung cancer: An independent prognostic factor after adjustment for clinical and dosimetric variables. RCR Open.

[40] Watanabe Y., Tomoda T., Iwata A., Matsuno H., Hayakawa H., Nagata T. (2026). Impact of heart dose on survival in palliative radiotherapy for bone metastases: Propensity score-matched analysis. In Vivo.

[41] Alcorn S., Cortés Á.A., Bradfield L., Brennan M., Dennis K., Diaz D.A., Doung Y.-C., Elmore S., Hertan L., Johnstone C. (2024). External beam radiation therapy for palliation of symptomatic bone metastases: An ASTRO clinical practice guideline. Pract. Radiat. Oncol..

[42] van der Linden Y.M., Steenland E., van Houwelingen H.C., Post W.J., Oei B., Marijnen C.A.M., Leer J.W.H. (2006). Patients with a favourable prognosis are equally palliated with single and multiple fraction radiotherapy: Results on survival in the Dutch Bone Metastasis Study. Radiother. Oncol..

[43] Chou Y.C., Lin C.Y., Pai P.C., Tseng C.K., Hsieh C.E., Chang K.P., Hsu C., Liao C., Wang C., Chin S. (2017). Dose-escalated radiation therapy is associated with better overall survival in patients with bone metastases from solid tumors: A propensity score-matched study. Cancer Med..

[44] Kao J., Farrugia M.K., Frontario S., Zucker A., Copel E., Loscalzo J., Sangal A., Darakchiev B., Singh A., Missios S. (2021). Association of radiation dose intensity with overall survival in patients with distant metastases. Cancer Med..

[45] Lewis T.S., Kennedy J.A., Price G.J., Mee T., Woolf D.K., Bayman N.A., Chan C., Coote J., Faivre-Finn C., Harris M. (2020). Palliative lung radiotherapy: Higher dose leads to improved survival?. Clin. Oncol..

[46] Krishnan M.S., Epstein-Peterson Z., Chen Y.H., Tseng Y.D., Wright A.A., Temel J.S., Catalano P., Balboni T.A. (2014). Predicting life expectancy in patients with metastatic cancer receiving palliative radiotherapy: The TEACHH model. Cancer.

[47] Chow E., Abdolell M., Panzarella T., Harris K., Bezjak A., Warde P., Tannock I. (2008). Predictive model for survival in patients with advanced cancer. J. Clin. Oncol..

[48] Rades D., Haus R., Schild S.E., Janssen S. (2019). Prognostic factors and a new scoring system for survival of patients irradiated for bone metastases. BMC Cancer.

