# Supplementary material for: Explainable Deep Learning Model for Predicting Overall Survival in Patients Receiving Palliative Radiotherapy for Bone Metastases
Source: Curr Oncol. 2026 Sep 2;33(9):528. doi: 10.3390/curroncol33090528 (PMC13605896; doi:10.3390/curroncol33090528)
Supplement: Supplementary file 1 [file curroncol-33-00528-s001.zip › curroncol-4489755-supplementary Table S1.pdf]

Supplementary Materials

Supplementary Table S1. Predictor variables and coding

Variable	Definition	Coding
ECOG PS	Performance status at the start of RT	1 = PS 0–1; 2 = PS 2; 3 = PS 3–4 (ordinal)
Age	Age at the start of RT	Years (continuous)
Sex	Biological sex	1 = male; 0 = female
Primary tumor risk group	Low risk = breast, prostate, hematologic; high risk = all others	1 = high risk; 0 = low risk
Multiple bone metastases	Number of bone metastases	1 = ≥ 2; 0 = solitary
Number of extraosseous sites	Count of involved extraosseous organ systems (0–7)	Integer (continuous)
Brain / lung / liver / non-regional lymph node / adrenal metastases; pleural / peritoneal dissemination	Presence of the respective extraosseous involvement	1 = present; 0 = absent (7 separate indicators)
Planned dose	Planned prescribed dose of the index course, converted to BED10 ($\alpha/\beta = 10$)	Gy (continuous)

There were no missing values in any variable. All inputs were z-standardized within each training fold. RT, radiation therapy; BED10, biologically effective dose with $\alpha/\beta = 10$; ECOG PS, Eastern Cooperative Oncology Group Performance Status.

Supplementary Code. The complete analysis pipeline (model training and validation, patient-level bootstrap confidence intervals, calibration, multivariable Cox analysis, SHAP and SurvLIME explainability, and sensitivity analyses) is provided as Supplementary File S1_Code.py, with tested library versions listed in its header.
